# Variability in instructions for performance of nasopharyngeal swabs across Canada in the era of COVID-19 – what type of swab is actually being performed?

**DOI:** 10.1186/s40463-020-00490-x

**Published:** 2021-01-28

**Authors:** Nole M. Hiebert, Breanna Ashley Chen, Leigh J. Sowerby

**Affiliations:** 1 Schulich School of Medicine and Dentistry, Western University, London, Ontario Canada; 2 Department of Pediatrics, Western University, London, Ontario Canada; 3 Department of Otolaryngology – Head and Neck Surgery, Western University, 1151 Richmond Street, London, Ontario N6A 3K7 Canada

**Keywords:** COVID-19, SARS-CoV-2, Nasopharyngeal swab, Turbinate, Nasopharynx, Screening

## Abstract

**Background:**

The primary method of surveillance for the presence of SARS-CoV-2 is with nasopharyngeal swabs. Given the significant demand for nasopharyngeal swabs, a large number of previously untrained and unfamiliar staff are now performing this test. It was noted that there was significant heterogeneity in instructions for performing nasopharyngeal swabs in Canada, in contrast to the guidance provided by the Centers for Disease Control and Prevention (CDC), and the Pan American Health Organization (PAHO). The objective of this study was to review the instructions provided across Canada and contrast them to those of the CDC and PAHO.

**Methods:**

A standard series of steps for nasopharyngeal swab performance was outlined based on the CDC, PAHO, and New England Journal of Medicine instructions. A comprehensive search was performed in August 2020 to identify nasopharyngeal swab guidelines provided by public health in the provinces and territories of Canada. Regional health authority guidance was also collected. Instructions provided were contrasted against the standardized steps.

**Results:**

Instructions were identified for all provinces and territories, and for 81 regional health authorities. From the provincial and territorial guidelines, 10/13 (77%) cleared the nasal passages before swab insertion, 11/13 (85%) tilted the patient’s head back slightly, 12/13 (92%) inserted the swab parallel to the palate, but only 3/13 (23%) inserted the swab to at least a depth of two-thirds the distance between the patient’s nose and ear. A clear majority (81%) of regional health authority guidelines followed their respective provincial guidelines.

For depth of insertion, Quebec provided a pictogram but no distance or technique for estimation. Six provinces and territories - Northwest Territories, Nunavut, Ontario, Saskatchewan, Prince Edward Island and Alberta - recommended 4 cm or one-half the distance from nostrils to ear. British Columbia and Manitoba recommended a 7 cm depth of insertion. Nova Scotia recommended one-half to two-thirds the distance from nose to ear. Lastly, Newfoundland, New Brunswick and the Yukon recommended an insertion from nose to the external ear canal.

**Conclusion:**

There is significant heterogeneity in guidance for nasopharyngeal swab performance across Canada. The instructions provided by the majority of provinces and territories in Canada would not be effective in reaching the nasopharynx.

**Graphical abstract:**

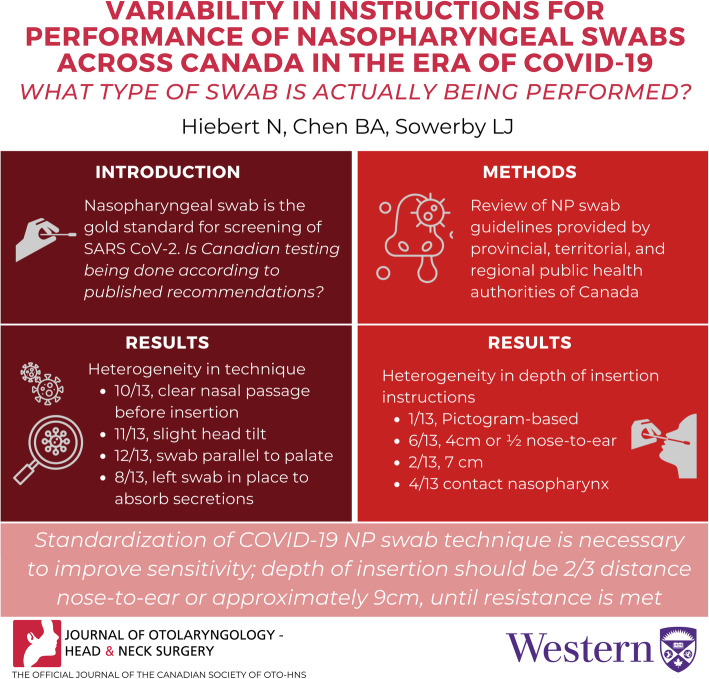

**Supplementary Information:**

The online version contains supplementary material available at 10.1186/s40463-020-00490-x.

## Introduction

Initial reports of a novel coronavirus disease 2019 (COVID-19) first came from Wuhan, Hubei Province of China in late December 2019 [1]. The causative agent, severe acute respiratory syndrome coronavirus 2 (SARS-CoV-2), has since spread rapidly around the world, with over 34 million cases reported as of October 2020. The highly infectious nature of SARS-CoV-2 and the presence of presymptomatic [2] and asymptomatic [3] viral shedding poses challenges to containing spread of the virus. The World Health Organization has identified high-volume testing of those at-risk and contact tracing as key pillars of ‘flattening the curve’ and suppressing future potential waves in the pandemic [4]. Identifying acute infection with SARS-CoV-2 virus is carried out via nucleic acid amplification testing (such as real-time reverse transcriptase polymerase-chain reaction) and specimens are most often obtained from the respiratory tract [5, 6].

Several studies have compared the effectiveness of nasopharyngeal swabs to oropharyngeal [7–9], nasal [7, 10, 11], and mid-turbinate [11, 12] specimens, with nasopharyngeal swabs being most sensitive. As such, the nasopharyngeal swab is currently considered the gold standard for screening specimen collection of SARS-CoV-2 [5–7, 10, 12, 13] and remains the recommended site of collection by the Canadian Public Health Laboratory Network Best Practices for COVID-19 [14]. Additionally, the nasopharyngeal swab is also the preferred specimen for influenza detection, which can have a similar clinical presentation and is expected to be circulating in the community simultaneously.

This review contrasts guidance documents from across Canada for performing nasopharyngeal swabs with instructions endorsed by the Centers for Disease Control and Prevention (CDC) [15], the Pan American Health Organization (PAHO) [16], and published in the New England Journal of Medicine (NEJM) [17]. Instructions from the CDC, PAHO, and NEJM are expert-informed and congruent with accepted means of reaching the nasopharynx and performing a nasopharyngeal swab [5, 6, 13].

## Methods

A comprehensive search strategy was performed in August 2020 to collect nasopharyngeal specimen collection guidelines from national, provincial, and territorial public health agencies, and from regional health authorities within each Canadian province and territory. Guidelines were acquired by navigating to publicly available COVID-19 resources for healthcare professionals posted on public health websites or via a Google search using keywords “COVID-19 guidance”, “nasopharyngeal specimen”, “nasopharyngeal collection”, “upper respiratory collection”, paired with the name of the respective health authority. If the guideline document was not found for a regional health authority, the assumption was made that the provincial guideline was being used if one of the following criteria were met:

i) the regional health authority COVID-19 resources specifically referenced the provincial guideline for nasopharyngeal specimen collection.
ii) the regional health authority referenced the general provincial COVID-19 resources, or;
iii) no publicly available COVID-19 resources for healthcare professionals were present on the regional health authority website.

To facilitate the comparison between the health authority guidelines and the CDC, PAHO, and NEJM guidelines, a combined CDC, PAHO, and NEJM guideline was first created. The steps in the three guidelines were highly congruent, except for the following:

i) The NEJM and PAHO guidelines included an initial step instructing the patient to blow their nose to clear the nasal passages.
ii) The CDC instructed to “tilt the patient’s head back 70°”, whereas the NEJM stated to “tilt the patient’s head back slightly.” The PAHO demonstrated tilting the patient’s head back slightly but did not state to what extent.
iii) All guidelines indicated that the correct depth of swab insertion was the approximate distance between the patient’s nose and ear, however the CDC and PAHO also indicated that the correct swab depth is when resistance is met.
iv) The CDC and NEJM instructed that the swab should be removed slowly while rotating it, whereas the PAHO guideline did not specify swab removal instructions.

The following steps for collection of a nasopharyngeal specimen were used to compare with the Canadian health authority recommendations. These steps were also congruent with other published protocols for nasopharyngeal swab performance [5, 6, 13, 18]:

1. Clear the nasal passages from excess mucous.
2. Tilt the patient’s head back slightly (around 70^o^ from the horizontal plane).
3. Insert the swab at an angle parallel to the palate along the nasal floor.
4. The swab should be inserted to at least a depth of approximately equal to two-thirds to the full distance between the patient’s nose and ear and stopped when resistance is met.
5. Rotate the swab back and forth.
6. Leave the swab in place for several seconds to absorb secretions.
7. Remove the swab while rotating it.
8. Place tip of swab into sterile viral transport media tube and snap/cut off the applicator stick.

## Results

Each of the 10 provinces and three territories had publicly accessible guidelines. All guidelines were written for their specific province or territory, except for Northwest Territories who are using the Alberta provincial guidelines. The province of New Brunswick referenced the PAHO guideline [19], however they also published an influenza-specific document with a different set of instructions for performing a nasopharyngeal swab [20]. Aside from New Brunswick, no other provincial guideline specifically referenced the CDC, PAHO, or NEJM guideline, nor did any follow all steps outlined by the CDC, PAHO, or NEJM. Each step of the combined CDC, PAHO, and NEJM guideline was compared to the respective step in each provincial and territorial guideline (Table 1). 10/13 (77%) cleared the nasal passages before swab insertion, 11/13 (85%) tilted the patient’s head back slightly, 12/13 (92%) inserted the swab parallel to the palate, 3/13 (23%) inserted the swab to at least a depth of two-thirds the distance between the patient’s nose and ear, 13/13 (100%) rotated the swab, 8/13 (62%) left the swab in place to absorb secretions, 1/13 (8%) rotated the swab while slowly removing it, and 13/13 (100%) handled the swab by placing the tip into a sterile viral transport media tube and snapping off or cutting the applicator stick.

**Table 1:** Comparison of provincial and territorial health authority guidelines to the combined CDC, PAHO, and NEJM guidelines

	Combined CDC and NEJM Guideline for Collection of Nasopharyngeal Specimen							
	Clear Nasal Passages	Head Placement	Angle of Swab Insertion	Depth of Swab Insertion	Swab Rotation	Swab Left in Place	Swab Removal	Swab Handling
Alberta [21]	✓	✓	✓		✓			✓
British Columbia [22]	✓	✓	✓		✓	✓		✓
Manitoba [23]	✓	✓	✓		✓	✓		✓
New Brunswick [19]	✓	✓	✓	✓	✓	✓		✓
Newfoundland and Labrador [24]	✓	✓	✓	✓	✓	✓		✓
Northwest Territories [21]	✓	✓	✓		✓			✓
Nova Scotia [25]	✓	✓	✓	✓	✓	✓		✓
Nunavut [26]	✓	✓	✓		✓			✓
Ontario [27]		✓	✓		✓	✓	✓	✓
Prince Edward Island [28]	✓	✓	✓		✓	✓		✓
Quebec [29]					✓			✓
Saskatchewan [30]		✓	✓		✓	✓		✓
Yukon Territory [31]	✓		✓	✓	✓			✓

Clear nasal passages – patient is instructed to blow their nose or the provider clears excess mucous prior to the swab; Head placement – tilted slightly back (i.e. at 70°); Angle of swab insertion – Parallel to palate along nasal floor; Depth of swab insertion – equal to at least approximately two-thirds the distance between the patient’s nose and ear; Swab rotation – the swab should be rotated several times at correct depth; Swab left in place – swab should be left at the nasopharynx for several seconds to absorb secretions; Swab removal – swab should be rotated while it is slowly removed; Specimen handling - tip of swab is to be placed into sterile viral transport media tube and the applicator stick is snapped or cut off

Provincial guidelines demonstrated the most heterogeneity with recommendations for depth of swab insertion (Table 2). Quebec makes no recommendation on specific depth of insertion, but provides a pictograph showing the swab at the posterior nasopharynx. Six provinces and territories - Northwest Territories, Nunavut, Ontario, Saskatchewan, Prince Edward Island and Alberta - recommended 4 cm or one-half the distance from nostrils to ear - which is effectively a mid-nasal swab. British Columbia and Manitoba recommended a 7 cm depth of insertion, which would reach the posterior nasal cavity but not the nasopharynx. Nova Scotia recommended a depth of insertion of one-half to two-thirds the distance from nose to ear. Lastly, Newfoundland recommended insertion to two-thirds of the distance from nostril to ear, while New Brunswick and the Yukon recommended the distance from nostrils to the external ear canal - all of which would effectively reach the nasopharynx.

**Table 2:** Provincial differences in the depth of insertion of a nasopharyngeal swab as outlined by published provincial guidelines

Province	Depth of Insertion
Alberta [21]	$$ \raisebox{1ex}{$1$}\!\left/ \!\raisebox{-1ex}{$2$}\right. $$ distance or 4 cm
British Columbia [22]	7 cm in adults
Manitoba [23]	7 cm in adults
New Brunswick [19]	$$ \raisebox{1ex}{$2$}\!\left/ \!\raisebox{-1ex}{$3$}\right. $$ to full distance from nostril to ear
Newfoundland and Labrador [24]	$$ \raisebox{1ex}{$2$}\!\left/ \!\raisebox{-1ex}{$3$}\right. $$distance from nostril to ear
Northwest Territories [21]	$$ \raisebox{1ex}{$1$}\!\left/ \!\raisebox{-1ex}{$2$}\right. $$distance or 4 cm
Nova Scotia [25]	$$ \raisebox{1ex}{$1$}\!\left/ \!\raisebox{-1ex}{$2\ $}\right. $$- $$ \raisebox{1ex}{$2$}\!\left/ \!\raisebox{-1ex}{$3$}\right. $$ distance from nostril to ear
Nunavut [26]	$$ \raisebox{1ex}{$1$}\!\left/ \!\raisebox{-1ex}{$2$}\right. $$distance from nostril to ear
Ontario [27]	$$ \raisebox{1ex}{$1$}\!\left/ \!\raisebox{-1ex}{$2$}\right. $$distance from nostril to ear
Prince Edward Island [28]	$$ \raisebox{1ex}{$1$}\!\left/ \!\raisebox{-1ex}{$2$}\right. $$distance from nostril to ear
Quebec [29]	Pictogram of nasopharynx
Saskatchewan [30]	$$ \raisebox{1ex}{$1$}\!\left/ \!\raisebox{-1ex}{$2$}\right. $$distance from nostril to ear
Yukon Territory [31]	Distance from nose to ear

Nasopharyngeal swabs must be inserted to at least approximately $$ \raisebox{1ex}{$2$}\!\left/ \!\raisebox{-1ex}{$3$}\right. $$ the distance between the patient’s nose to ear to effectively sample the nasopharynx

Of the 81 regional health authorities across Canada, 66/81 (81%) used their respective provincial or territorial guideline. 2/81 (2.5%) either referenced the CDC, PAHO, or NEJM guidelines or included all of the outlined steps in their own guideline (Supplementary Table 1). Of interest, only 10/81 (12%) inserted the swab to at least a depth of two-thirds the distance between the patient’s nose and ear.

## Discussion

**Fig. 1:**
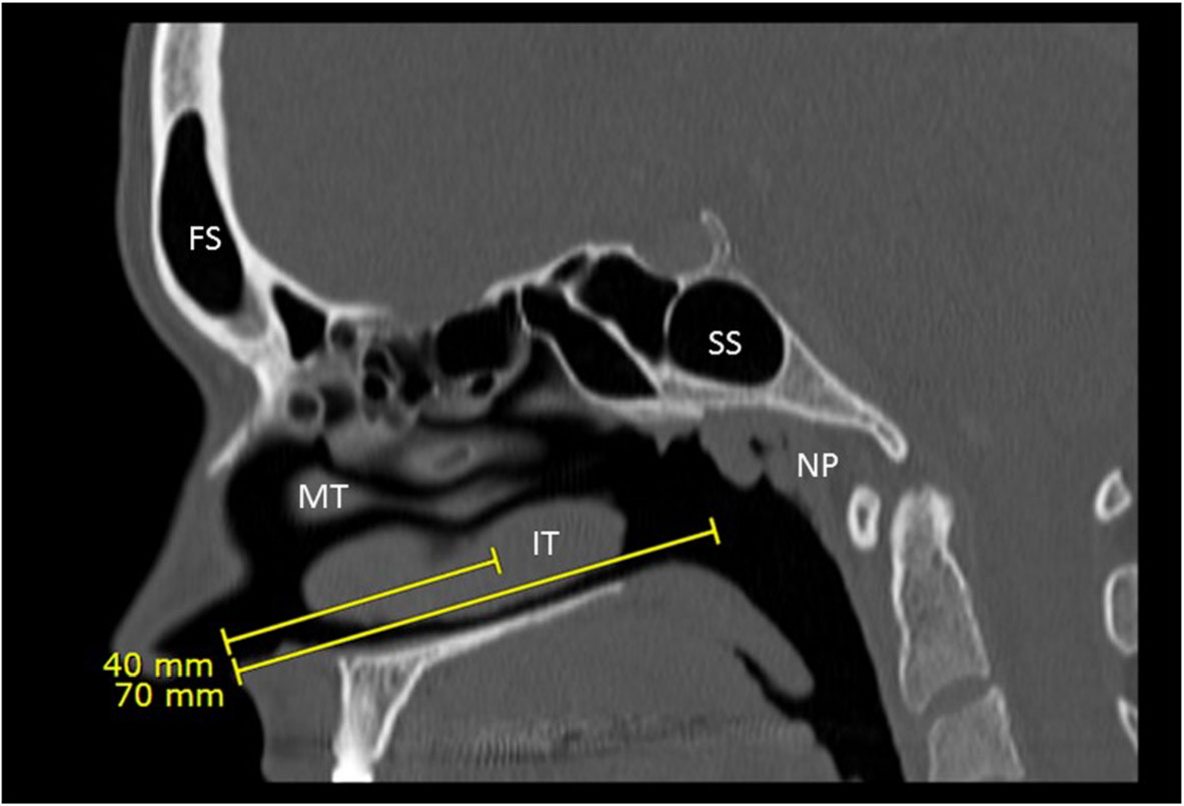
Sagittal CT view of nose and nasopharynx (50-year-old Caucasian female). Areas reached by recommended measured depth of insertion are demonstrated. FS - Frontal Sinus, MT - Middle Turbinate, IT - Inferior Turbinate, SS - Sphenoid Sinus, NP - Nasopharynx

This review has demonstrated significant heterogeneity in nasopharyngeal swab performance instructions across Canada. The bottom line is that a swab needs to be inserted to the appropriate depth in order to reach the nasopharynx if one is to perform a *nasopharyngeal* swab (Fig. 1, Supplemental video 1). As experts in regional anatomy of the head and neck, otolaryngologists can provide a great service by actively engaging with their local and regional health authorities to train proper technique and anatomical knowledge.

Minimal data has been published regarding the depth of the nasopharynx. A study by Lim and Lee [32] from Korea demonstrated in a series of 200 individuals that the average distance from nostril to nasopharynx surface was 9.4 ± 0.6 cm in females and 10.0 ± 0.5 cm in males. They also found that the average distance from philtrum to tragus (akin to measuring nostril to ear distance) in the same set of patients was 14.4 ± 0.5 cm in females and 15.1 ± 0.6 cm in males. A study examining 227 subjects demonstrated that the distance from nostril to face of sphenoid was 68 ± 4 mm in females and 71 ± 6 mm in males [33], which falls short of the nasopharynx. As such, in order to reach the nasopharyngeal mucosa, a swab should be inserted to a depth of two-thirds to the full distance from nostril to ear, or at least nine centimeters, and stopped when resistance is met.

There is some heterogeneity to the literature but, on the whole, it appears that swabs are more likely to detect SARS-CoV-2 in the nasopharynx than in other areas of the nasal cavity. Pinninti, Trieu [12] performed paired swabs over time in a series of 40 patients with COVID-19. They found that 85% of initial nasopharyngeal swabs were positive versus 73% for mid-turbinate swabs performed at the same time. They also found that nasopharyngeal swabs were more likely to be positive with lower viral loads, as demonstrated by higher cycle thresholds. Péré, Podglajen [10] performed a similar study comparing nasal swabs to nasopharyngeal swabs in 44 patients. Using the nasopharyngeal swab as the gold standard, only 75% of nasal swabs performed concurrently were positive. Tu, Jennings [11] reported on a series of over 500 patients in which multiple swabs were performed by patients on themselves and compared to a nasopharyngeal swab performed by a healthcare provider. Of the 51 patients positive for SARS-CoV-2 on either nasal or nasopharyngeal swab, 47 were positive on both, 3 were positive only on nasopharyngeal swab and 1 was only positive on nasal swab. In comparing mid-turbinate swabs to nasopharyngeal swabs, 50 were positive on both while 2 were positive only on nasopharyngeal swab. Interestingly, the cycle thresholds in this study were higher (indicating a lower viral load) for the mid-turbinate swab than the nasopharyngeal swab in 83% of cases.

The ideal sequence of steps in performing a nasopharyngeal swab has not been subjected to the scientific method, and likely never will be. Subtle variations in nasopharyngeal swab technique have not been evaluated and contrasted, and as such it is not possible to comment on whether certain aspects of swab performance are superior to others. As such, establishing a ‘gold standard’ for performance is challenging and will rely on expert consensus, anatomical knowledge, and experience. As more is learned from the outbreak of COVID-19, recommendations could change in favour of a different type of sample for SARS-CoV-2 testing. High-volume, low-cost testing - such as home saliva tests - may well be more important than high sensitivity testing in the future. Nevertheless, nasopharyngeal swabs will remain an important sampling method for other common respiratory viruses, such as influenza, further underscoring the need for a standardized approach across Canada.

## Conclusion

Diagnostic screening for SARS-CoV-2 during the COVID-19 pandemic is of paramount importance for source control and contact tracing. Nasopharyngeal swabs have, thus far, been demonstrated to be the preferred method of sample collection. This study has demonstrated that there is significant heterogeneity across Canada in instructions for performing a nasopharyngeal swab - and, most importantly, in depth of swab insertion. The depth of swab insertion directly determines the type of swab performed - if not inserted deep enough, a mid-nasal or mid-turbinate swab is performed instead, potentially reducing the sensitivity of the swab.

## Supplementary Information

**Additional file 1: Supplementary Table 1.** Comparison of regional health authority guidelines to the combined CDC and NEJM guideline.

**Additional file 2.**

## Data Availability

All data used for this manuscript is available on request. Previous versions of the final draft of this manuscript are also available for review.

## References

[1] Zhu N, Zhang D, Wang W, Li X, Yang B, Song J (2020). A novel coronavirus from patients with pneumonia in China, 2019. N Engl J Med.

[2] Byrne AW, McEvoy D, Collins AB, Hunt K, Casey M, Barber A (2020). Inferred duration of infectious period of SARS-CoV-2: rapid scoping review and analysis of available evidence for asymptomatic and symptomatic COVID-19 cases. BMJ Open.

[3] Gao Z, Xu Y, Sun C, Wang X, Guo Y, Qiu S, et al. A systematic review of asymptomatic infections with COVID-19. J Microbiol Immunol Infect. 2020. 10.1016/j.jmii.2020.05.001 PMC7227597 32425996

[4] Critical preparedness, readiness and response actions for COVID-19: Interim Guidance. World Health Organization; 2020.

[5] Mawaddah A, Gendeh HS, Lum SG, Marina MB (2020). Upper respiratory tract sampling in COVID-19. Malaysian J Pathol.

[6] Tang Y-W, Schmitz JE, Persing DH, Stratton CW (2020). Laboratory diagnosis of COVID-19: current issues and challenges. J Clin Microbiol.

[7] LeBlanc JJ, Heinstein C, MacDonald J, Pettipas J, Hatchette TF, Patriquin G. A combined oropharyngeal/nares swab is a suitable alternative to nasopharyngeal swabs for the detection of SARS-CoV-2. J Clin Virol. 2020;128. 10.1016/j.jcv.2020.104442 PMC7228872 32540034

[8] Wang X, Tan L, Wang X, Liu W, Lu Y, Cheng L (2020). Comparison of nasopharyngeal and oropharyngeal swabs for SARS-CoV-2 detection in 353 patients received tests with both specimens simultaneously. Int J Infect Dis.

[9] Wang D, Hu B, Hu C, Zhu F, Liu X, Zhang J, et al. Clinical Characteristics of 138 Hospitalized Patients With 2019 Novel coronavirus-infected pneumonia in Wuhan, China. JAMA 2020. 10.1001/jama.2020.1585 PMC7042881 32031570

[10] Péré H, Podglajen I, Wack M, Flamarion E, Mirault T, Goudot G (2020). Nasal swab sampling for SARS-CoV-2: a convenient alternative in times of nasopharyngeal swab shortage. J Clin Microbiol.

[11] Tu Y-P, Jennings R, Hart B, Cangelosi GA, Wood RC, Wehber K (2020). Swabs collected by patients or health care workers for SARS-CoV-2 testing. N Engl J Med.

[12] Pinninti S, Trieu C, Pati SK, et al. Comparing Nasopharyngeal and Mid-Turbinate Nasal Swab Testing for the Identification of SARS-CoV-2 [published online ahead of print, 2020 Jun 29]. Clin Infect Dis. 2020;ciaa882. 10.1093/cid/ciaa882.

[13] Pondaven-Letourmy S, Alvin F, Boumghit Y, Simon F. How to perform a nasopharyngeal swab in adults and children in the COVID-19 era. Eur Ann Otorhinolaryngol Head Neck Dis. 2020;137(4):325–7. 10.1016/j.anorl.2020.06.001. 10.1016/j.anorl.2020.06.001 PMC7274641 32646750

[14] Respiratory Virus Infections Working G (2020). Canadian public health laboratory network best practices for COVID-19. Can Commun Dis Rep.

[15] Interim Guidelines for Collecting, Handling, and Testing Clinical Specimens for COVID-19 [Web page]. Centers for Disease Control and Prevention; 2020 [updated July 8, 2020. Available from: https://www.cdc.gov/coronavirus/2019-ncov/lab/guidelines-clinical-specimens.html#:~:text=Swab%20should%20reach%20depth%20equal,remove%20swab%20while%20rotating%20it.

[16] Nasopharyngeal and Oropharyngeal Swabs [Web page]. Pan American Health Organization; 2012 [Available from: https://www.paho.org/hq/index.php?option=com_content&view=article&id=7918:2012-videos-sample-collection&Itemid=40295&lang=pt.

[17] Marty FM, Chen K, Verrill KA (2020). How to obtain a nasopharyngeal swab specimen. N Engl J Med.

[18] Petruzzi G, De Virgilio A, Pichi B, Mazzola F, Zocchi J, Mercante G (2020). COVID-19: nasal and oropharyngeal swab. Head Neck.

[19] COVID-19: Guidance for Long-Term Care Facilities (LTCF). New Brunswick: Office of the Chief Medical Officer of Health; 2020 [updated May 4, 2020. Available from: https://www2.gnb.ca/content/dam/gnb/Departments/h-s/pdf/covid-19_ltcf_guidance-e.pdf.

[20] New Brunswick Sentinel Practitioner Influena Network (NB-SPIN) (2018). Lab Specimen Submission. The Government of New Brunswick.

[21] Collection of a Nasopharyngeal Swab for Detection of Respiratory Infection Alberta: Alberta Health Services; 2020 [updated May 2020. Available from: https://www.albertahealthservices.ca/assets/wf/plab/wf-provlab-collection-of-nasopharyngeal-and-throat-swab.pdf.

[22] COVID-19: Adult Viral Testing Guidelines for British Columbia [PDF]. British Columbia: BC Centre for Disease Control; 2020 [updated August 7, 2020. Available from: http://www.bccdc.ca/Health-Professionals-Site/Documents/BCCDC_PHL_Updated_nCoV_Lab_Guidance.pdf.

[23] Standard Operating Procedure (SOP) for: Nasopharyngeal Swab (NP) [PDF]. Manitoba: SharedHealth Manitoba; 2020 [updated March 18, 2020. Available from: https://sharedhealthmb.ca/files/covid-19-sop-swab.pdf.

[24] Nasopharyngeal Swabs for COVID-19 Virus and Other Respiratory Pathogen Testing [PDF]. Newfoundland and Labrador: Provincial Public Health Laboratory Network; 2020 [updated April 16, 2020. Available from: https://publichealthlab.ca/wp-content/uploads/2020/04/PHML-Swab-Collection-UPDATED-REVISED-Memorandum-April-16-2020-FOR-PROVINCE-WIDE-DISTRIBUTION.pdf.

[25] Nasopharyngeal Swab Collection and Screening for Respiratory Illness [PDF]. Nova Scotia: Nova Scotia Health Authority; 2019 [updated January 2, 2019. Available from: http://policy.nshealth.ca/Site_Published/nsha/document_render.aspx?documentRender.IdType=6&documentRender.GenericField=&documentRender.Id=71770.

[26] Nunavut Communicable Disease Manual [PDF]. Nunavut: Government of Nunavut; 2020 [updated April 2020. Available from: https://gov.nu.ca/sites/default/files/covid-19_public_health_protocol_v4_20apr2020.pdf.

[27] Virus Respiratory Kit (Nasopharyngeal) Kit Order #: 390082 [URL]. Ontario: Public Health Ontario; 2020 [updated July 23, 2020. Available from: https://www.publichealthontario.ca/en/laboratory-services/kit-test-ordering-instructions/virus-respiratory-kit.

[28] Guide to Laboratory Services [PDF]. Prince Edward Island: Health PEI; 2014 [updated September 15, 2014. Available from: http://www.gov.pe.ca/photos/original/hpei_labguide.pdf.

[29] Guide de Prélèvement pour Dépistage COVID-19 [PDF]. Québec: Institut National de Santé Publique; 2020 [updated May 5, 2020. Available from: https://www.inspq.qc.ca/sites/default/files/lspq/guide-prelevement-ecouvillon-hors-norme-milieu-maison.pdf.

[30] Specimen Collection and Laboratory Testing for COVID-19 [PDF]. Saskatchewan: Saskatchewan Health Authority; 2020 [updated April 23, 2020. Available from: https://www.saskatchewan.ca/government/health-care-administration-and-provider-resources/treatment-procedures-and-guidelines/emerging-public-health-issues/2019-novel-coronavirus/information-for-health-care-providers/testing-screening-treatment-and-medical-directives/physician-memo.

[31] COVID-19 Testing Recommendations [PDF]. Yukon: Government of Yukon; 2020 [updated March 20, 2020. Available from: http://www.hss.gov.yk.ca/pdf/npswab.pdf.

[32] Lim H, Lee JH, Son KK, Han YJ, Ko S (2014). A method for optimal depth of the nasopharyngeal temperature probe: the philtrum to tragus distance. Korean J Anesthesiol.

[33] Mohamadi Y, Mousavi M, Pakzad R, Hassanzadeh G (2016). Anthropometric parameters for access to Sella Turcica through the nostril. J Craniofac Surg.

