# Supplementary material for: Variability in instructions for performance of nasopharyngeal swabs across Canada in the era of COVID-19 – what type of swab is actually being performed?
Source: J Otolaryngol Head Neck Surg. 2021 Jan 28;50:5. doi: 10.1186/s40463-020-00490-x (PMC7841972; doi:10.1186/s40463-020-00490-x)
Supplement: Supplementary file 1 — Additional file 1: Supplementary Table 1. Comparison of regional health authority guidelines to the combined CDC and NEJM guideline. [file 40463_2020_490_MOESM1_ESM.docx]

**Supplementary Material**

| Supplementary Table 1: Comparison of regional health authority guidelines to the combined CDC and NEJM guideline | | | | | | | | | | |
| --- | --- | --- | --- | --- | --- | --- | --- | --- | --- | --- |
|  | **Province** | **Provincial Guideline in Use** | **Combined CDC and NEJM Guideline for Collection of Nasopharyngeal Specimen** | | | | | | | |
|  |  |  | **Clear Nasal Passages** | **Head Placement** | **Angle of Swab Insertion** | **Depth of Swab Insertion** | **Swab Rotation** | **Swab Left in Place** | **Swab Removal** | **Swab Handling** |
| North Zone (1) | AB | Yes | ✓ | ✓ | ✓ |  | ✓ |  |  | ✓ |
| Edmonton Zone (1) | AB | Yes | ✓ | ✓ | ✓ |  | ✓ |  |  | ✓ |
| Central Zone (1) | AB | Yes | ✓ | ✓ | ✓ |  | ✓ |  |  | ✓ |
| Northern Health Authority (2) | BC | Yes | ✓ | ✓ | ✓ |  | ✓ | ✓ |  | ✓ |
| Interior Health Authority (3) | BC | No |  | ✓ | ✓ |  | ✓ | ✓ |  | ✓ |
| Vancouver Island Health Authority (2) | BC | Yes | ✓ | ✓ | ✓ |  | ✓ | ✓ |  | ✓ |
| Vancouver Coastal Health Authority (4) | BC | No | ✓ | ✓ | ✓ |  | ✓ | ✓ | ✓ | ✓ |
| Fraser Health Authority (5) | BC | No |  |  | ✓ |  | ✓ | ✓ |  | ✓ |
| First Nations Health Authority (6) | BC | No | ✓ |  | ✓ | ✓ | ✓ | ✓ |  | ✓ |
| Provincial Health Services Authority (2) | BC | Yes | ✓ | ✓ | ✓ |  | ✓ | ✓ |  | ✓ |
| Prairie Mountain Health (7) | MB | Yes | ✓ | ✓ | ✓ |  | ✓ | ✓ |  | ✓ |
| Interlake Eastern Regional Health Authority (7) | MB | Yes | ✓ | ✓ | ✓ |  | ✓ | ✓ |  | ✓ |
| Southern Health-Sante Sud (7) | MB | Yes | ✓ | ✓ | ✓ |  | ✓ | ✓ |  | ✓ |
| Northern Regional Health Authority (7) | MB | Yes | ✓ | ✓ | ✓ |  | ✓ | ✓ |  | ✓ |
| Winnipeg Regional Health Authority (7) | MB | Yes | ✓ | ✓ | ✓ |  | ✓ | ✓ |  | ✓ |
| Vitalité Health Network (8) | NB | Yes | ✓ | ✓ | ✓ | ✓ | ✓ | ✓ |  | ✓ |
| Horizon Health Network (8) | NB | Yes | ✓ | ✓ | ✓ | ✓ | ✓ | ✓ |  | ✓ |
| Grenfell Regional Health Services Board (9) | NL | Yes | ✓ | ✓ | ✓ | ✓ | ✓ | ✓ |  | ✓ |
| Health and Community Services Central Region (9) | NL | Yes | ✓ | ✓ | ✓ | ✓ | ✓ | ✓ |  | ✓ |
| Health and Community Services Eastern Region (9) | NL | Yes | ✓ | ✓ | ✓ | ✓ | ✓ | ✓ |  | ✓ |
| Health and Community Services Western Region (9) | NL | Yes | ✓ | ✓ | ✓ | ✓ | ✓ | ✓ |  | ✓ |
| Western Zone (10) | NS | Yes | ✓ | ✓ | ✓ |  | ✓ | ✓ |  | ✓ |
| Northern Zone (10) | NS | Yes | ✓ | ✓ | ✓ |  | ✓ | ✓ |  | ✓ |
| Central Zone (10) | NS | Yes | ✓ | ✓ | ✓ |  | ✓ | ✓ |  | ✓ |
| Eastern Zone (10) | NS | Yes | ✓ | ✓ | ✓ |  | ✓ | ✓ |  | ✓ |
| Beaufort-Delta HSS Authority (1) | NT | Yes | ✓ | ✓ | ✓ |  | ✓ |  |  | ✓ |
| Sahtu HSS Authority (1) | NT | Yes | ✓ | ✓ | ✓ |  | ✓ |  |  | ✓ |
| Deh Cho HSS Authority (1) | NT | Yes | ✓ | ✓ | ✓ |  | ✓ |  |  | ✓ |
| Tlicho HSS Authority (1) | NT | Yes | ✓ | ✓ | ✓ |  | ✓ |  |  | ✓ |
| Yellowknife HSS Authority (1) | NT | Yes | ✓ | ✓ | ✓ |  | ✓ |  |  | ✓ |
| Stanton Territorial Health Authority (1) | NT | Yes | ✓ | ✓ | ✓ |  | ✓ |  |  | ✓ |
| Hay River HSS Authority (1) | NT | Yes | ✓ | ✓ | ✓ |  | ✓ |  |  | ✓ |
| Fort Smith HSS Authority (1) | NT | Yes | ✓ | ✓ | ✓ |  | ✓ |  |  | ✓ |
| Nunavut Health Region (11) | NU | Yes | ✓ | ✓ | ✓ |  | ✓ |  |  | ✓ |
| Erie St. Clair LHIN (12) | ON | No |  | ✓ |  |  | ✓ | ✓ | ✓ | ✓ |
| South West LHIN (12) | ON | No |  | ✓ |  |  | ✓ | ✓ | ✓ | ✓ |
| Waterloo Wellington LHIN (13) | ON | Yes |  | ✓ | ✓ |  | ✓ | ✓ | ✓ | ✓ |
| Hamilton Niagara Haldimand Brant LHIN (13) | ON | Yes |  | ✓ | ✓ |  | ✓ | ✓ | ✓ | ✓ |
| Central West LHIN (13) | ON | Yes |  | ✓ | ✓ |  | ✓ | ✓ | ✓ | ✓ |
| Mississauga Halton LHIN (13) | ON | Yes |  | ✓ | ✓ |  | ✓ | ✓ | ✓ | ✓ |
| Toronto Central LHIN (13) | ON | Yes |  | ✓ | ✓ |  | ✓ | ✓ | ✓ | ✓ |
| Central LHIN (13) | ON | Yes |  | ✓ | ✓ |  | ✓ | ✓ | ✓ | ✓ |
| Central East LHIN (13) | ON | Yes |  | ✓ | ✓ |  | ✓ | ✓ | ✓ | ✓ |
| South East LHIN (13) | ON | Yes |  | ✓ | ✓ |  | ✓ | ✓ | ✓ | ✓ |
| Champlain LHIN (13) | ON | Yes |  | ✓ | ✓ |  | ✓ | ✓ | ✓ | ✓ |
| North Simcoe Muskoka LHIN (13) | ON | Yes |  | ✓ | ✓ |  | ✓ | ✓ | ✓ | ✓ |
| North East LHIN (14) | ON | No |  | ✓ | ✓ | ✓ | ✓ | ✓ | ✓ | ✓ |
| North West LHIN (13) | ON | Yes |  | ✓ | ✓ |  | ✓ | ✓ | ✓ | ✓ |
| Health PEI (15) | PE | Yes | ✓ | ✓ | ✓ |  | ✓ | ✓ |  | ✓ |
| Région de l’Abitibi-Témiscamingue (16) | QC | No |  | ✓ |  |  | ✓ | ✓ |  | ✓ |
| Région de l’Estrie (17) | QC | No | ✓ | ✓ | ✓ |  | ✓ | ✓ |  | ✓ |
| Région de l’Outaouais (18) | QC | Yes |  |  |  |  | ✓ |  |  | ✓ |
| Région de la Capitale-Nationale (18) | QC | Yes |  |  |  |  | ✓ |  |  | ✓ |
| Région de la Chaudière-Appalaches(19) | QC | No |  | ✓ | ✓ | ✓ | ✓ | ✓ |  | ✓ |
| Région de la Côte-Nord (18) | QC | Yes |  |  |  |  | ✓ |  |  | ✓ |
| Région de la Gaspésie-Îles-de-la-Madeleine (20) | QC | No |  | ✓ |  |  | ✓ |  |  | ✓ |
| Région de la Mauricie et du Centre-du-Québec (18) | QC | Yes |  |  |  |  | ✓ |  |  | ✓ |
| Région de la Montérégie (18) | QC | Yes |  |  |  |  | ✓ |  |  | ✓ |
| Région de Lanaudière (18) | QC | Yes |  |  |  |  | ✓ |  |  | ✓ |
| Région de Laval (21) | QC | No |  | ✓ |  |  | ✓ | ✓ |  | ✓ |
| Région de Montréal-Centre (20) | QC | No |  | ✓ |  |  | ✓ |  |  | ✓ |
| Région des Laurentides (18) | QC | Yes |  |  |  |  | ✓ |  |  | ✓ |
| Région des Terres-Cries-de-la-Baie-James (18) | QC | Yes |  |  |  |  | ✓ |  |  | ✓ |
| Région du Bas-Saint-Laurent (16) | QC | No |  |  |  |  | ✓ | ✓ |  | ✓ |
| Région du Nord-du-Québec (18) | QC | Yes |  |  |  |  | ✓ |  |  | ✓ |
| Région du Nunavik (22) | QC | No | ✓ | ✓ | ✓ |  | ✓ | ✓ |  | ✓ |
| Région du Saguenay - Lac-Saint-Jean (18) | QC | Yes |  |  |  |  | ✓ |  |  | ✓ |
| Athabasca Health Authority (23) | SK | Yes |  | ✓ | ✓ |  | ✓ | ✓ |  | ✓ |
| Cypress (23) | SK | Yes |  | ✓ | ✓ |  | ✓ | ✓ |  | ✓ |
| Five Hills (23) | SK | Yes |  | ✓ | ✓ |  | ✓ | ✓ |  | ✓ |
| Heartland (23) | SK | Yes |  | ✓ | ✓ |  | ✓ | ✓ |  | ✓ |
| Keewatin Yatthé (23) | SK | Yes |  | ✓ | ✓ |  | ✓ | ✓ |  | ✓ |
| Kelsey Trail (23) | SK | Yes |  | ✓ | ✓ |  | ✓ | ✓ |  | ✓ |
| Mamawetan Churchill River (23) | SK | Yes |  | ✓ | ✓ |  | ✓ | ✓ |  | ✓ |
| Prairie North (23) | SK | Yes |  | ✓ | ✓ |  | ✓ | ✓ |  | ✓ |
| Prince Albert Parkland (23) | SK | Yes |  | ✓ | ✓ |  | ✓ | ✓ |  | ✓ |
| Regina Qu’Appelle (23) | SK | Yes |  | ✓ | ✓ |  | ✓ | ✓ |  | ✓ |
| Saskatoon (23) | SK | Yes |  | ✓ | ✓ |  | ✓ | ✓ |  | ✓ |
| Sun Country (23) | SK | Yes |  | ✓ | ✓ |  | ✓ | ✓ |  | ✓ |
| Sunrise (23) | SK | Yes |  | ✓ | ✓ |  | ✓ | ✓ |  | ✓ |
| Yukon Territory Health Region (24) | YT | Yes | ✓ |  | ✓ | ✓ | ✓ |  |  | ✓ |

Clear nasal passages – patient is instructed to blow their nose or the provider clears excess mucous prior to the swab; Head placement – tilted slightly back (i.e. at 70°); Angle of swab insertion – Parallel to palate along nasal floor; Depth of swab insertion – equal to at least approximately 2/3 distance between the patient’s nose and ear; Swab rotation – the swab should be rotated several times at correct depth; Swab left in place – swab should be left at the nasopharynx for several seconds to absorb secretions; Swab removal – swab should be rotated while it is slowly removed; Specimen handling - tip of swab is to be placed into sterile viral transport media tube and the applicator stick is snapped or cut off.

**Regional Health Authority References**

1. Collection of a Nasopharyngeal Swab for Detection of Respiratory Infection Alberta: Alberta Health Services; 2020 [updated May 2020. Available from: <https://www.albertahealthservices.ca/assets/wf/plab/wf-provlab-collection-of-nasopharyngeal-and-throat-swab.pdf>.

2. COVID-19: Adult Viral Testing Guidelines for British Columbia [PDF]. British Columbia: BC Centre for Disease Control; 2020 [updated August 7, 2020. Available from: <http://www.bccdc.ca/Health-Professionals-Site/Documents/BCCDC_PHL_Updated_nCoV_Lab_Guidance.pdf>.

3. COVID-19 Interim Guidance: Nasopharyngeal Viral Swab Collection [PDF]. British Columbia: Interior Health Authority; 2020 [updated May 2020. Available from: <https://www.interiorhealth.ca/YourEnvironment/CommunicableDiseaseControl/covid19/IH-PH-COV-500%20COVID-19%20Nasopharyngeal%20Viral%20Swab%20Collection%20UTM%20Swab%20Kit_Interim%20Guidance.pdf>.

4. How to use Guide: Nasopharyngeal Flocked Swabs and Universal Transport Medium (UTM) [URL]. British Columbia: Vancouver Coastal Health Authority; 2020 [Available from: <http://ipac.vch.ca/Pages/Emerging-Issues.aspx>.

5. COVID-19 Binder: Response Guidance for Long-Term Care, Assisted Living and Independent Living Facilities [PDF]. British Columbia: Fraser Health Authority; 2020 [updated May 6, 2020. Available from: <https://www.fraserhealth.ca/-/media/Project/FraserHealth/FraserHealth/Health-Professionals/Clinical-resources/COVID19/LTC_AL_IL-COVID-binder_May6.pdf>.

6. Clinical Procedure: Nasopharyngeal Swab Testing [PDF]. British Columbia: First Nations Health Authority; 2020 [updated July 22, 2020. Available from: <https://www.fnha.ca/Documents/FNHA-Clinical-Procedure-Nasopharyngeal-Swab-Testing.pdf>.

7. Standard Operating Procedure (SOP) for: Nasopharyngeal Swab (NP) [PDF]. Manitoba: SharedHealth Manitoba; 2020 [updated March 18, 2020. Available from: <https://sharedhealthmb.ca/files/covid-19-sop-swab.pdf>.

8. COVID-19: Guidance for Long-Term Care Facilities (LTCF) [PDF]. New Brunswick: Office of the Chief Medical Officer of Health; 2020 [updated May 4, 2020. Available from: <https://www2.gnb.ca/content/dam/gnb/Departments/h-s/pdf/covid-19_ltcf_guidance-e.pdf>.

9. Nasopharyngeal Swabs for COVID-19 Virus and Other Respiratory Pathogen Testing [PDF]. Newfoundland and Labrador: Provincial Public Health Laboratory Network; 2020 [updated April 16, 2020. Available from: <https://publichealthlab.ca/wp-content/uploads/2020/04/PHML-Swab-Collection-UPDATED-REVISED-Memorandum-April-16-2020-FOR-PROVINCE-WIDE-DISTRIBUTION.pdf>.

10. Nasopharyngeal Swab Collection and Screening for Respiratory Illness [PDF]. Nova Scotia: Nova Scotia Health Authority; 2019 [updated January 2, 2019. Available from: <http://policy.nshealth.ca/Site_Published/nsha/document_render.aspx?documentRender.IdType=6&documentRender.GenericField=&documentRender.Id=71770>.

11. Nunavut Communicable Disease Manual [PDF]. Nunavut: Government of Nunavut; 2020 [updated April 2020. Available from: <https://gov.nu.ca/sites/default/files/covid-19_public_health_protocol_v4_20apr2020.pdf>.

12. COVID-19 Testing Policy and Guidelines [PDF]. London, Ontario: London Health Science Centre Pathology and Laboratory Medicine; 2020 [updated August 20, 2020. Available from: <https://www.lhsc.on.ca/palm/docs/collection%20guide.pdf>.

13. Virus Respiratory Kit (Nasopharyngeal) Kit Order #: 390082 [URL]. Ontario: Public Health Ontario; 2020 [updated July 23, 2020. Available from: <https://www.publichealthontario.ca/en/laboratory-services/kit-test-ordering-instructions/virus-respiratory-kit>.

14. Interim Guidelines for Collecting, Handling, and Testing Clinical Specimens for COVID-19 [Web page]. Centers for Disease Control and Prevention; 2020 [updated July 8, 2020. Available from: <https://www.cdc.gov/coronavirus/2019-ncov/lab/guidelines-clinical-specimens.html#:~:text=Swab%20should%20reach%20depth%20equal,remove%20swab%20while%20rotating%20it>.

15. Guide to Laboratory Services [PDF]. Prince Edward Island: Health PEI; 2014 [updated September 15, 2014. Available from: <http://www.gov.pe.ca/photos/original/hpei_labguide.pdf>.

16. Prélèvement du spécimen de dépistage du SARS-CoV-2 (COVID-19) [PDF]. Québec: Centre Intégré de Santé et de Services Sociaux du Bas-Saint-Laurent; 2020 [updated May 25, 2020. Available from: <https://www.cisss-bsl.gouv.qc.ca/sites/default/files/f-m-334-1_prelevement_du_specimen_de_depistage_du_sars-cov-2_covid-19.pdf>.

17. Prélèvement par écouvillonnage nasopharyngé et oropharyngé pour le dépistage de la COVID-19 [PDF]. Québec: Centre Intégré Universitaire de Santé et de Services Sociaux de l’Estrie - Centre Hospitalier Universitaire de Sherbrooke; 2020 [updated May 19, 2020. Available from: <https://www.santeestrie.qc.ca/clients/SanteEstrie/Conseils-sante/Infections-maladies/COVID-19/MSI-AS-001_prelevement_secretion_nasopharynge_et_oropharynge2020-05-20.pdf>.

18. Guide de Prélèvement pour Dépistage COVID-19 [PDF]. Québec: Institut National de Santé Publique; 2020 [updated May 5, 2020. Available from: <https://www.inspq.qc.ca/sites/default/files/lspq/guide-prelevement-ecouvillon-hors-norme-milieu-maison.pdf>.

19. COVID-19 Communication aux médecins [PDF]. Québec: Centre Intégre de Santé et de Services Sociaux de Chaudière-Appalaches; 2020 [updated June 3, 2020. Available from: <https://www.cisssca.com/clients/CISSSCA/CISSS/COVID-19/Medecins/NS_%C3%A9couvillons_COVID19_2020-06-03.pdf>.

20. Prélèvement des échantillons nasopharyngés par écouvillonage [Web page]. CHU Sainte-Justine; 2020 [updated March 17, 2020. Available from: <https://vimeo.com/398248118/3f51f7bae0>.

21. Procédure de prélèvement oropharyngé et nasopharyngé et transport du spécimen au laboratoire [Web page]. Centre Intégré de Santé et de Services Sociaux de Laval; 2020 [updated March 23, 2020. Available from: <https://www.youtube.com/watch?v=2eHsKvobqhs>.

22. Prélèvement des sécrétions des voies respiratoires supérieures [PDF]. Québec: Régie Régionale de la Santé et des Services Sociaux du Nunavik; 2020 [updated May 26, 2020. Available from: <https://nrbhss.ca/sites/default/files/baocovid/MSI_Prelevement_des_secretions_des_voies-respiratoires_sup_2020-04-22.pdf>.

23. Specimen Collection and Laboratory Testing for COVID-19 [PDF]. Saskatchewan: Saskatchewan Health Authority; 2020 [updated April 23, 2020. Available from: <https://www.saskatchewan.ca/government/health-care-administration-and-provider-resources/treatment-procedures-and-guidelines/emerging-public-health-issues/2019-novel-coronavirus/information-for-health-care-providers/testing-screening-treatment-and-medical-directives/physician-memo>.

24. COVID-19 Testing Recommendations [PDF]. Yukon: Government of Yukon; 2020 [updated March 20, 2020. Available from: <http://www.hss.gov.yk.ca/pdf/npswab.pdf>.
